# Sleep quality among emergency nurses and its influencing factors during COVID-19 pandemic: a cross-sectional study

**DOI:** 10.3389/fpsyg.2024.1363527

**Published:** 2024-07-19

**Authors:** Awatif Alrasheeday, Maha Ali Alsaeed, Bushra Alshammari, Farhan Alshammari, Asia Saad Alrashidi, Turki Ahmed Alsaif, Soha Kamel Mahmoud, Dolores I. Cabansag, Ma Venus Borja, Ahmad R. Alsayed, Omaima Mohamed Elalem, Shaimaa Mohamed Nageeb, Rania Abd-Elnaby Allam, Tahani Nasser Alhejaili, Haneen Fahad Alsulami, Bahia Galal Abd Elrazik Siam

**Affiliations:** ^1^Department of Nursing Administration, College of Nursing, University of Hail, Hail, Saudi Arabia; ^2^Damam Health Network, Eastern Health Cluster, Dammam, Saudi Arabia; ^3^Department of Medical Surgical Nursing, College of Nursing, University of Hail, Hail, Saudi Arabia; ^4^Department of Pharmaceutics, College of Pharmacy, University of Hail, Hail, Saudi Arabia; ^5^Department of Mental Health Nursing, College of Nursing, University of Hail, Hail, Saudi Arabia; ^6^Community Health Nursing, College of Nursing, University of Hail, Hail, Saudi Arabia; ^7^Department of Community Health Nursing, Faculty of Nursing, Benha University, Banha, Egypt; ^8^Department of Clinical Pharmacy and Therapeutics, Applied Science Private University, Amman, Jordan; ^9^Family and Community Health Nursing, Faculty of Nursing, Port Said University, Port Said, Egypt; ^10^Department of Maternal and Child, College of Nursing, University of Hail, Hail, Saudi Arabia; ^11^Adult ICU, Maternity and Children Hospital, Makkah Health Cluster, Makkah, Saudi Arabia; ^12^Adult ICU, King Abdullah Medical City Hospital, Makkah Health Cluster, Makkah, Saudi Arabia; ^13^Department of Medical Surgical Nursing, Faculty of Nursing, Port Said University, Port Said, Egypt

**Keywords:** emergency nurses, factors, sleep quality, COVID-19, sleep disturbances

## Abstract

**Background:**

COVID-19 has probably contributed to sleep disturbance among nurses, especially those working at emergency departments (EDs). Sleep disorders in nurse managers can negatively impact their health and impair work performance and decision-making. This study aimed to explore the quality of sleep among nurses working in EDs and its influencing factors during the COVID-19 pandemic.

**Method:**

In this study, a cross-sectional design was employed to assess the sleep quality of nurses working in EDs during the COVID-19 pandemic. The research recruited a convenience sample of emergency nurses, who were selectively sourced from four hospitals in Hail City. This recruitment occurred over the period from April to July 2022. Descriptive data analysis was conducted using SPSS, with the significance level set at 0.05.

**Results:**

Among the 216 participants in the study, the majority (55.6%) were aged between 30 and 39 years, and 73.6% were female. Additionally, 64.4% were married, while 69.4% had a bachelor’s degree, 20% held a diploma, and the remaining had a master’s degree. Notably, a significant 81.5% of the nurses reported poor sleep quality, as assessed by the Pittsburgh Sleep Quality Index (PSQI), with an overall mean score of 10.55 indicating poor sleep. The study highlighted that poor sleep quality among nurses was linked to being female, being married, and not exercising regularly. Better sleep was associated with nurses who manage fewer patients per shift and have adequate monthly income.

**Conclusion:**

The prevalence of poor sleep quality among Emergency nurses during the COVID-19 pandemic is high. There is a pressing need for targeted interventions to enhance sleep quality among ED nurses. Improving sleep quality is not only essential for the wellbeing of these nurses but is also likely to contribute to better patient care.

## Introduction

The COVID-19 pandemic, which emerged as a global health crisis in late 2019, profoundly impacted health worldwide. By early 2022, the World Health Organization (WHO) reported a significant rise in global COVID-19 cases, with a sharp increase in reported cases, totaling approximately 23.5 million weekly (World Health Organization, 2024). The COVID-19 pandemic heightened stress, anxiety, and uncertainty due to increased cases and hospitalizations. These factors significantly impact sleep, causing disturbances, disrupted routines, and changes in work environments, particularly affecting frontline workers like nurses in Eds (Madkhali et al., 2022; Guerrera et al., 2024). The surge in patient numbers overwhelmed healthcare systems, especially in EDs, increasing the workload for nurses. This resulted in longer, irregular hours and disrupted sleep patterns, leading to sleep problems, anxiety, and depression (Pollard et al., 2020).

Quality sleep is crucial for nurses in high-stress environments like EDs; it is essential for maintaining physical and mental wellbeing, cognitive function, and overall quality of life (Abraham et al., 2017; Zeng et al., 2020). Nurses regularly face numerous work-related pressures, including heavy workloads, the need to manage interactions with patients and their families, shift work, and overtime (Bae and Fabry, 2014; Alshammari et al., 2022). Sleep problems among nurses, including insomnia, insufficient sleep, deprivation, and poor quality, are prevalent, with rates ranging from 57 to 83% (Qiu et al., 2020) and up to 50% worldwide experience short sleep durations (Furihata et al., 2020; Stimpfel et al., 2020). Low-quality sleep will not only affect their health and wellbeing but also has significant implications for patient care and safety as it can impair cognitive functions such as memory, attention, and decision-making, which are critical in the fast-paced and often high-stakes environment of an ED.

The unique stressors of the COVID-19 pandemic, including the fear of virus contraction, treating severely ill patients, and witnessing suffering, contribute to increased stress and anxiety among ED nurses (Fawaz et al., 2020). The pandemic also alters ED working conditions, with added complexities like personal protective equipment (PPE) and infection control protocols, impacting nurses’ physical exhaustion and sleep quality (Fawaz et al., 2020; Hoedl et al., 2021). The nature of shift work, especially night shifts inherent in emergency nursing, significantly affects the sleep quality of ED nurses. This, combined with rotating schedules, can disrupt circadian rhythms, leading to shift work sleep disorder. The pandemic’s demand for extended and irregular shifts further exacerbates these disruptions, impacting sleep quality (Dong et al., 2020).

Several factors influence their sleep quality, including gender, marital status, exercise habits, monthly income, and patient load (Krueger and Friedman, 2009). Female nurses often report poorer sleep quality due to the additional societal and domestic responsibilities they bear, which increase their stress levels (Almeida et al., 2020). Marital status also impacts sleep quality, with various statuses such as being single, married, divorced, or widowed influencing stress and sleep patterns differently (Troxel et al., 2007). Regular physical exercise is known to improve sleep quality by reducing stress and anxiety (Kredlow et al., 2015). Financial stability, indicated by higher monthly income, is associated with better sleep quality due to reduced financial stress and access to comfortable living conditions (Du et al., 2021). Lastly, the number of patients a nurse is responsible for during a shift significantly affects their stress levels, which in turn impacts sleep quality, with higher patient loads leading to increased stress and poorer sleep (Aiken et al., 2002). These factors, particularly in the context of nurses, need to be thoroughly explored to understand their impact on sleep quality. Poor sleep quality not only affects nurses directly but also has broader implications for healthcare systems and patient care. Studies show that sleep issues can reduce productivity, potentially lowering nursing care standards (Dong et al., 2017). Sleep-deprived nurses may struggle to maintain attention and vigilance, crucial for patient safety and effective care delivery (Stimpfel et al., 2020), increasing the risk of errors, a significant concern in EDs (Wardle-Pinkston et al., 2019).

The research aims to provide valuable insights into the pandemic’s impact on healthcare workers, specifically focusing on the sleep quality of ED nurses. It seeks to inform strategies for supporting their health and wellbeing by addressing the challenges they face during the pandemic (Alshammari et al., 2023). Additionally, the study aims to contribute to the broader discourse on healthcare worker support during crises, emphasizing the necessity of systemic changes to ensure their wellbeing, considering their integral role in healthcare systems.

Aim of the study: This study aimed to explore the quality of sleep among nurses working in EDs and its influencing factors during the COVID-19 pandemic.

Research questions:

1. What is the level of sleep quality among nurses working in the EDs during COVID-19 Pandemic?
2. What are the factors influencing sleep quality among nurses working in the EDs during COVID-19 Pandemic?

Research hypothesis

H_1_: Female gender has a significant association with lower sleep quality among nurses working in the ED during the COVID-19 pandemic.

H_2_: Being married has a significant association with lower sleep quality among nurses working in the ED during the COVID-19 pandemic.

H_3_: Regular physical exercise is positively associated with the sleep quality of nurses working in the ED during the COVID-19 pandemic.

H_4_: Monthly income is positively associated with the sleep quality of nurses working in the ED during the COVID-19 pandemic.

H_5_: The number of patients a nurse is responsible for during a night shift is negatively associated with sleep quality.

## Subjects and methods

### Study design

A cross-sectional descriptive study design was employed to assess the sleep quality and its influencing factors among nurses working in ED during the COVID-19 pandemic. All staff nurses working in the EDs were invited to participant in this research. Data collection took place between April and July 2022.

### Setting

The sample was recruited from four government hospitals in Hail City, namely King Salman Specialist Hospital, Sharaf Hospital, Hail General Hospital, and King Khaled Hospital. These selected hospitals are government-run medical facilities that offer complimentary services and emergency treatment exclusively to Saudi nationals facing health issues. Each of these hospitals has the capacity to accommodate hundreds of patients across various specialties, including oncology, cardiology, obesity, and neurology. Additionally, they are equipped with Intensive Care Units (ICUs) and specialized units for burns, endoscopy, surgery, day surgery, and physiotherapy. Furthermore, they house various specialized and support service sections to cater to a broad spectrum of healthcare needs.

### Sampling and population

In this study, we approached a convenience sample of 330 nurses. Of these, 216 ED nurses completed the survey. Eligibility for participation was limited to registered nurses who were actively employed on a full-time basis in the EDs, had a minimum of one year of work experience, and expressed their willingness to participate in the study. The study excluded nurses who were not regular staff of the ED but were only covering shifts in the ED, in addition to newly hired nurses and intern nurses.

### Data collection

In the selected hospitals, gatekeepers, who were the head nurses, played a crucial role in identifying eligible nurses for the study. These gatekeepers were responsible for distributing the participation link, generated via Google Forms, to these selected nurses. This link led to a page where the nurses found an invitation to participate, along with an information sheet and a consent form. Upon providing their consent, the nurses gained access to the survey. They could then complete and submit the questionnaire at their convenience. The questionnaire takes 10 min to complete.

To ensure data accuracy in the questionnaires, all items were set as required to guarantee response completeness. Additionally, each IP address or device was limited to a single submission to prevent duplicate responses, thus maintaining the integrity and reliability of the data.

### Tools for data collections

An English language, self-administered questionnaire was utilized to collect data, consisting of four parts:

Part I: This section gathers the sociodemographic data of the study subjects, including gender, age, educational level, marital status, and the number of children in the household.
Part II: This part focuses on professional and work-related information, including monthly income, professional rank, number of work hours per week, number of night shifts per month, and the number of patients under their care during a shift.
Part III: This section addressed daily activities related to the sleep quality of the nurses, including questions about smoking status, tea/coffee consumption, and exercise habits.
Part IV: This section incorporates the Pittsburgh Sleep Quality Index (PSQI), an English version adopted from Buysse et al. (1989). The PSQI is a self-rated scale used to assess the nurses’ sleep quality and sleep disturbances over a one-month interval. The PSQI has nineteen items make up seven sub-dimensions: subjective sleep quality, sleep latency, sleep duration, habitual sleep efficacy, sleep disturbance, use of sleep medication, and daytime dysfunction, each scored 0 (no difficulty) to 3 (severe difficulty). The component scores are summed to produce a global score (range 0 to 21). Higher scores indicate poor sleep quality. A systematic review and meta-analysis study confirmed that the PSQI demonstrated strong reliability and validity, as well as intermediate structural validity, in a variety of samples (Mollayeva et al., 2016). This indicates that the tool serves its intended purpose (Aiken et al., 2002). Nurses who scored five or more on the PSQI global score were classified as having poor sleep, while those who scored less than five were judged to have normal sleep.

### Data analysis

The collected data were tabulated and statistically analyzed using the statistical package for social science (SPSS) advanced statistics, version 26. Continuous variables were presented using mean and standard deviation (SD), while categorical variables were expressed as frequency and percentage. *t*-tests and one-way analysis of variance (ANOVA) were used to examine the association between sleep quality mean scores and categorical variables with two groups (*t*-test) or more than two groups ANOVA. Multiple linear regression was used for multivariate analyses with the PSQI as the dependent variable, including only significant independent variables in the model and excluding non-significant ones. All tests were conducted at the 0.05 level of statistical significance. In cases of missing data, any responses with incomplete data, whether entirely or partially missing, were excluded from the analyses.

### Ethical considerations

Ethical approval for the study was obtained from the Institutional Review Board (IRB), represented by the Health Cluster in Hail city [registered with the King Abdullah City for Science and Technology (KACS) in the KSA, under the registration number H-08-L-074, with Approval No: 2022-23]. Informed consent was obtained from all participants prior to their engagement in the study. In this research, every participant was briefed on the study’s aims and benefits before they filled out the questionnaire. Their freedom to choose to be part of the study was assured, and confidentiality of their information was guaranteed, with usage limited strictly to research purposes. The survey used were anonymous, ensuring they couldn’t be linked back to the individuals who filled them. The survey didn’t include any identifying details like names or contact info. To enhance the protection of participants’ anonymity, all responses were collected, merged, and then summarized in the final report.

## Results

Table 1 shows that most of the respondents (55.6%) aged between 30 and 39 years old, with an overall mean (SD) of 33.1 (6.6). A higher proportion were females (73.6%), married (64.4%), had bachelor’s degrees (69.4%), almost two-thirds of the nurses (42.1%) earned a monthly salary of 6,000–8,000 Saudi Riyal and 47.2% of them were having one to three dependents. Regarding their working status, the results show that the majority of nurses were registered nurses (56.5%), 46.3 of them had night shifts at least 7 times a month, 47.7 % worked for at least 45 h a week, and 56.9 % of them had less than 30 patients admitted per shift. As regards the health-related factors; 44.0 % of the studied nurses had normal BMI, 77.3 % of them took tea and coffee daily, slightly more than half practice smoking daily (50.9%). However, 6.9% of them never smoke, and 58.8% of them were sometimes practicing exercise.

**TABLE 1:** Percentage distribution of the studied nurses based on their sociodemographic, work status, and health-related factors (N. 216).

Variables	Frequency	Percentage (%)
**Age**		
• 20–29	68	31.5
• 30–39	120	55.6
• ≥ 40	28	12.9
**Mean (SD): 33.1 (6.6)**		
**Gender**		
• Male	57	26.4
• Female	159	73.6
**Marital status**		
• Single	63	29.2
• Married	139	64.4
• Divorced/widowed	14	6.5
**Educational level**		
• Diploma	45	20.8
• Bachelor	150	69.4
• Master or above	21	9.7
**Monthly income**		
• 6,000–8,000 SR	91	42.1
• 8,000–10,000 SR	49	22.7
• > 10,000	76	35.2
**Number of dependents**		
• None	92	42.6
• 1–3	102	47.2
• 4–7	22	10.2
**Professional rank**		
• Senior	41	19
• Intermediate	7	3.2
• Registered	122	56.5
• Primary	14	6.5
• Years of experience	32	14.8
**Night shift frequency per month**		
• Never	57	26.4
• 1–3	21	9.7
• 4–6	38	17.6
• > 7	100	46.3
**Work hours/week**		
• 40	18	8.3
• 40–45	95	44
• > 45	103	47.7
**Number of patients admitted/shift.**		
• < 30	123	56.9
• 30–60	67	31
• > 60	26	12
**Body mass index**		
• Underweight	8	3.7
• Normal	95	44.0
• Overweight	79	36.6
• Obese	34	15.7
**Tea/coffee consumption daily/almost daily**		
• Often	167	77.3
• Sometimes	34	15.7
• Never/almost never	15	6.9
**Smoking status**		
• Daily/almost daily	110	50.9
• Often	35	16.2
• Sometimes	57	26.4
• Never/almost never	15	6.9
**Practicing exercise**		
• Often	29	13.4
• Sometimes	127	58.8
• Never/almost never	60	27.8

SD, standard deviation.

Table 2 reveals that highest mean scores of the PSQI components were for Component 5 (sleep disturbances), Component 1 (subjective sleep quality), Component 3 (sleep duration), and Component 4 (habitual sleep efficiency), with a global mean score of 10.55. It was also notable that the score for the use of sleep medications among nurses is relatively low (1.44), suggesting a relatively infrequent reliance on pharmacological aids for sleep. The mean score for daytime disfunction was 0.42, indicating relatively high efficiency of daily work among the participants even with poor sleep quality.

**TABLE 2:** Mean scores of the Pittsburgh Sleep Quality Index (PSQI).

Components of PSQI	Normal		Poor		Mean 8	SD
	N.	%	N.	%		
1. Subjective sleep quality	21	9.7	195	90.3	1.92	0. 32
2. Sleep latency	137	63.4	79	36.6	0.97	0.69
3. Sleep duration	82	38.0	134	62.0	1.74	0.92
4. Habitual sleep efficiency	84	38.9	132	61.1	1.61	1.34
5. Sleep disturbance	39	18.1	177	81.9	2.46	1.07
6. Using sleep medications	129	59.7	87	40.3	1.44	0.71
7. Daytime dysfunction	190	88.0	26	12.0	0.42	0.82

Global mean of PSQI: 10.55 ± 3.30.

**FIGURE 1:**
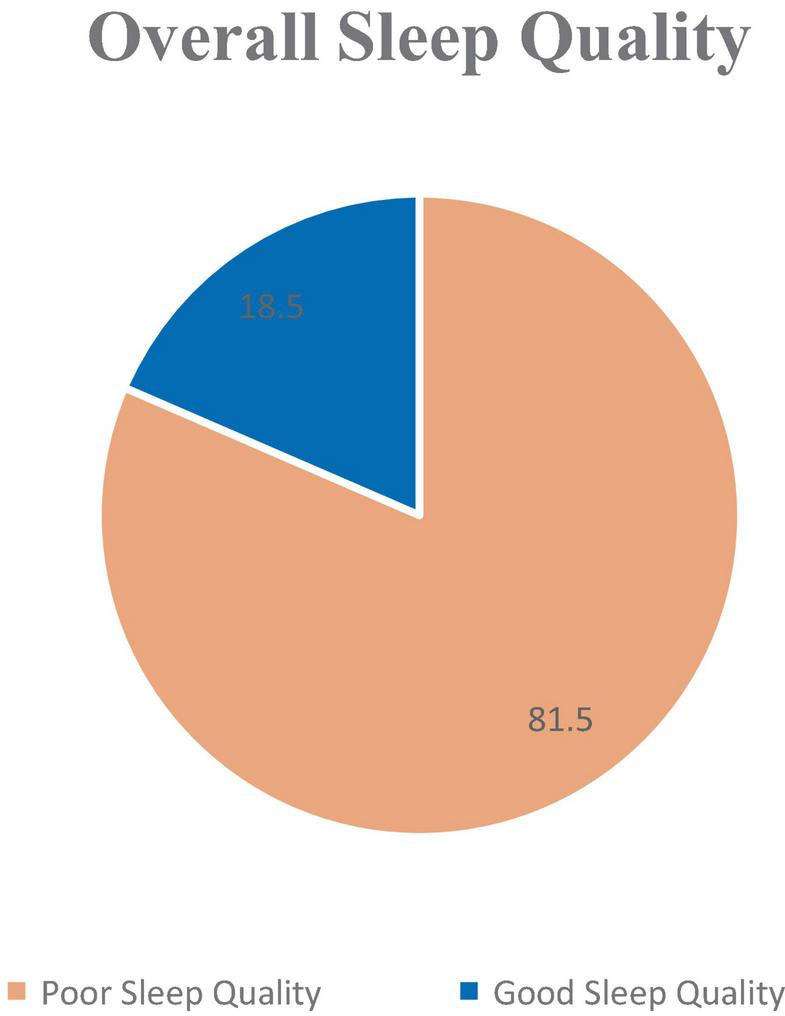
The overall PSQI level of the respondents.

Figure 1 illustrates that 81.5% of the participants were categorized as having poor sleep quality according to the PSQI scale. Conversely, only 18.5% of them demonstrated normal sleep quality.

As shown in Table 3, the overall mean scores of the PSQI were significantly associated with gender, marital status, monthly income, patient load per shift, and practicing exercise. Female nurses, those who had moderate income, who had higher number of admitted patients per shift, and those who never practice exercise had higher mean PSQI scores indicative of poorer sleep quality compared to other categories (*p*-value < 0.05). However, there were no significant differences in sleep quality across other variables.

**TABLE 3:** Relationship between total mean scores of PSQI and some variables.

Variables	Total PSQI Mean ± SD	Test of Sig.	*p*-value
**Age**			
• 20–29	10.53 ± 3.48	*F* = 1.87	0.339
• 30–39	10.63 ± 3.33		
• ≥ 40	10.29 ± 2.84		
**Mean (SD): 33.1 (6.6)**			
**Gender**			
• Male	10.19 ± 3.29	*t* = 2.064	0.009*
• Female	11.58 ± 3.15		
**Marital status**			
• Single	9.86 ± 3.55	*F* = 3.093	0.047*
• Married	10.91 ± 3.16		
• Divorced/widowed	10.21 ± 3.33		
**Educational level**			
• Diploma	10.44 ± 3.23	*F* = 0.323	0.724
• Bachelor	11.20 ± 3.30		
• Master or above	10.00 ± 3.78		
**Monthly income**			
• 6,000–8,000 SR	9.48 ± 2.30	*F* = 4.377	0.014*
• 8,000–10,000 SR	11.63 ± 3.57		
• > 10,000	11.14 ± 3.14		
**Number of dependents**			
• None	10.13 ± 3.34	*F* = 1.634	0.198
• 1–3	10.71 ± 3.31		
• 4–7	11.50 ± 3.02		
**Professional rank**			
• Senior	11.66 ± 3.17	*F* = 1.164	0.328
• Intermediate	12.14 ± 4.84		
• Registered	10.11 ± 3.09		
• Primary	11.57 ± 3.52		
• Years of experience	10.03 ± 3.49		
**Night shift frequency per month**			
• Never	10.30 ± 3.46	*F* = 1.389	0.247
• 1–3	11.14 ± 3.02		
• 4–6	11.32 ± 2.81		
• > 7	10.29 ± 3.43		
**Work hours/week**			
• 40	9.28 ± 4.01	*F* = 0.583	0.559
• 40–45	10.61 ± 3.17		
• > 45	1,073 ± 3.28		
**Number of patients admitted/shift**			
• < 30	9.72 ± 3.31	*F* = 5.816	0.003*
• 30–60	11.46 ± 3.01		
• > 60	12.19 ± 2.87		
**Tea/coffee consumption**			
• Daily/almost daily	11.00 ± 4.19	*F* = 0.778	0.461
• Often	10.24 ± 3.11		
• Sometimes	10.50 ± 3.39		
• Never/almost never			
**Smoking status**			
• Daily/almost daily	10.72 ± 3.44	*F* = 1.680	0.189
• Often	9.41 ± 2.87		
• Sometimes	10.74 ± 3.31		
• Never/almost never			
**Body mass index**			
• Underweight	8.13 ± 4.64	*F* = 0.179	0.673
• Normal	10.63 ± 3.27		
• Overweight	10.73 ± 3.14		
• Obese	10.50 ± 3.39		
**Practicing exercise**			
• Often	10.72 ± 3.44	*F* = 4.333	0.014*
• Sometimes	9.41 ± 2.87		
• Never/almost never	10.74 ± 3.31		

*F*, one-way ANOVA, *T*, *t*-test,

*P: statistically significant at *p* ≤ 0.05.

Table 4 Presents that The PSQI of the studied subjects was best predicted by gender, income, and workload (number of patients in the charge of night shift).

**TABLE 4:** Multiple linear regression analyses for some predictor variables and PSQI scores of the studied group.

Predictor variable	Unstandardized coefficients		Standardized coefficients	*T*	Sig.
	B	Std. error	Beta		
Gender	0.531	0.272	0.145	1.950	0.05
Marital status	−0.170	0.196	−0.058	−0.868	0.387
Exercise	−0.057	0.122	−0.031	−0.469	0.640
Monthly income	−0.359	0.141	−0.195	−2.542	0.012
Number of patients in the charge of a night shift	−0.548	0.172	−0.216	−3.196	0.002
Constant	4.258	0.772		5.518	0.000

Adjusted *R*^2^ = 0.106, *p* < 001. B, beta co-efficient; SEB, standard error.

## Discussion

This study aims to investigate the sleep quality of Saudi emergency nurses during the COVID-19 pandemic and identify the factors influencing it. Sleeping problems are common among nurses and could have a variety of unpleasant effects. A change in sleep quality might impair productivity and lower the standard of nursing care (Dong et al., 2020).

The findings of the current study showed that a majority of respondents (81.5%) experienced poor sleep quality (Figure 1), with a global mean PSQI score of 10.55 (Table 2), indicative of poor sleep quality among nurses. This finding is consistent with findings from other studies, including those by Furihata et al. (2020), Khatony et al. (2020), Qiu et al. (2020), Stimpfel et al. (2020), and Vadi et al. (2022), all of which reported the poor sleep quality is a common problem among nurses. Further supporting this, Dong et al. (2017) found that 64% of nurses reported sleep disturbances within the nursing profession.

The sleep quality among the nurses in this study was found to be associated with gender, as female nurses exhibiting higher instances of poor sleep quality. This observation aligns with the findings of Cattani et al. (2021), who also reported an association between sleep quality and gender. Similarly, Alharbi et al. (2021) noted that being female was linked to poorer sleep. Additionally, Yilmaz et al. (2021), concluded that female nurses were more prone to experiencing poor sleep quality. The observed findings can be interpreted through various ways. Female nurses often have to balance their professional duties with familial caregiving responsibilities (Yilmaz et al., 2021). This dual role can elevate stress levels and limit the time available for restful sleep (DePasquale et al., 2019). Another explanation is the hormonal changes associated with the menstrual cycle, pregnancy, and menopause can profoundly affect sleep patterns, as noted by Baker and Lee (2022). Societal and cultural expectations can influence sleep quality. In many cultures, women face additional pressures that can lead to increased stress and disrupted sleep (Knutson, 2013). Additionally, some studies have proposed that women are generally more likely than men to recognize and report problems associated with sleep disturbances (Knutson, 2013).

Furthermore, a link was found between sleep quality and monthly income, where emergency nurses earning lower salaries experienced more sleep difficulties. This finding aligns with Gao et al. (2022) research, which identified low monthly income as a contributing factor to poor sleep quality in most participants. Similarly, a study conducted in Jordan by Suleiman et al. (2020) established a positive correlation between the amount of salary and sleep quality, further emphasizing the impact of income on sleep. The findings can be attributed to several factors. Financial stress from lower income can increase anxiety and worry, adversely affecting sleep. Additionally, lower income often limits access to a conducive sleep environment, potentially resulting in less comfortable living conditions and exposure to disruptive factors like noise and inadequate temperature control (Johnson et al., 2018).

Besides personal factors, the study found that the quantity of patients a nurse managed per shift associated negatively with their sleep quality (the higher the number of patients, the higher PSQI scores and poor sleep). This aligns with findings from a French longitudinal cohort study by Badahdah et al. (2020), which connected sleep issues to both intensive labor and extended workdays. Supporting this, research by Dong et al. (2017) and Dong et al. (2020) suggests that primary and registered nurses are particularly prone to sleep disorders. Factors such as longer working hours and the patient load per shift were also identified as contributors to reduced sleep quality. Dealing with more patients, especially in an ED setting, often involves managing critical and high-stress situations. This can lead to emotional and mental fatigue, which makes it harder to unwind and achieve restful sleep.

Our study’s results show that sleep quality was not significantly associated with sociodemographic factors such as age, education level, and professional rank. This aligns with findings by Johnson et al. (2018) and Segon et al. (2022), who also reported no significant correlation between age, educational status, and sleep deprivation. In addition, Badahdah et al. (2020) and Wang et al. (2021) observed no substantial link between poor sleep quality and professional rank in their research.

The current findings indicate a statistically significant difference in the PSQI total scores among nurses in relation to their exercise habits. This might be due to the stress-reducing effects of exercise, which in turn could improve sleep quality. This observation is consistent with the studies by Strand et al. (2013) and Chen et al. (2022), which noted that the frequency of exercise positively influenced the sleep quality of nurses.

Our study’s results indicate that there was no significant association between poor sleep quality and habits like smoking or consuming tea and coffee. This contrasts with the findings of Strand et al. (2013), Omar et al. (2022), and Sayik et al. (2022), who observed that both smokers and tea/coffee drinkers tended to have lower overall PSQI-mean scores, suggesting poorer sleep quality. This difference in findings could be attributed to factors such as the timing and quantity of smoking or caffeine consumption. Specifically, consuming caffeine later in the day may have a more significant impact on sleep compared to morning intake. This variable, which can crucially affect sleep quality, might not have been consistently accounted for across different studies. Additionally, it’s noteworthy that our study did not measure these aspects, which could further explain the variation in results.

In relation to predictor variables of sleep quality, the present study showed that sleep quality among the studied subjects was best predicted by gender, income, and workload (number of patients in the charge of night shift), answering research question (2). These findings were in the same line with (Kim-Godwin et al., 2021), who pointed out that the nurses working full time (*t* = 2.41, *p* = 0.02), showed poor overall sleep quality. While these findings contradicted (Chen et al., 2022) who revealed that nurse managers’ sleep quality can be greatly affected by demographic characteristics such as their ages and exercise regularity.

This study has several limitations that should be considered. Firstly, the cross-sectional nature of the research design prevents the establishment of cause-and-effect relationships. Secondly, focusing on a specific government institution in the northern region of Saudi Arabia raises concerns regarding selection bias, thus limiting the generalizability of the findings. Additionally, exclusively including emergency nurses, may not accurately reflect the experiences of nurses in other specialties or departments, cautioning against extrapolating these findings to a broader nursing population. Furthermore, the study did not account for some potentially relevant factors, such as the psychological impact of the COVID-19 pandemic, individual health conditions, or personal coping mechanisms, which might influence sleep quality. Moreover, there is a possibility that some observed disturbances may be normative rather than solely attributable to the pandemic. Factors beyond the pandemic, such as societal stressors and individual circumstances, may contribute to sleep disruptions during the study period. Therefore, we encourage future research to explore these complexities further, ensuring a nuanced understanding of sleep disturbances in the context of both pandemics and broader societal influences. Finally, the study failed to explore the influence of COVID-related stress on sleep quality among nurses. This overlooks a crucial aspect of their wellbeing, particularly in high-stress environments like EDs during the pandemic. Future research should examine this association to provide a more comprehensive understanding of nurses’ sleep patterns.

## Conclusion

The study reveals a high prevalence of poor sleep quality among emergency nurses during the COVID-19 pandemic. Factors such as gender, marital status, monthly income, the number of patients managed per shift, and exercise habits significantly associated with poor sleep quality in this group. These findings underline the multifaceted nature of sleep disturbances in a high-stress healthcare environment and emphasize the need for targeted interventions to address these issues. Healthcare institutions should consider implementing policies and programs aimed at improving sleep quality among nurses. This could include scheduling reforms to reduce overwork, stress reduction workshops, and health promotion activities that encourage exercise. Creating a support system within the workplace to address the unique challenges faced by different demographic groups, such as female nurses or those with lower incomes, is also crucial.

## Data availability statement

The raw data supporting the conclusions of this article will be made available by the authors, without undue reservation.

## Ethics statement

Ethical approval for the study was obtained from the Institutional Review Board (IRB), represented by the Health Cluster in Hail city [registered with the King Abdullah City for Science and Technology (KACS) in the KSA, under the registration number H-08-L-074, with Approval No: 2022-23]. The studies were conducted in accordance with the local legislation and institutional requirements. The participants provided their written informed consent to participate in this study.

## Author contributions

AwA: Writing – original draft, Writing – review & editing. MA: Writing – original draft, Writing – review & editing. BA: Conceptualization, Data curation, Formal analysis, Funding acquisition, Investigation, Methodology, Project administration, Resources, Software, Supervision, Validation, Visualization, Writing – original draft, Writing – review & editing. FA: Writing – original draft, Writing – review & editing. AsA: Writing – original draft, Writing – review & editing. TAA: Writing – original draft, Writing – review & editing. SM: Writing – original draft, Writing – review & editing. DC: Writing – original draft, Writing – review & editing. MB: Writing – original draft, Writing – review & editing. AhA: Writing – original draft, Writing – review & editing. OE: Writing – original draft, Writing – review & editing. SN: Writing – original draft, Writing – review & editing. RA: Writing – original draft, Writing – review & editing. TNA: Writing – original draft, Writing – review & editing. HA: Writing – original draft, Writing – review & editing. BS: Data curation, Formal analysis, Investigation, Methodology, Writing – original draft, Writing – review & editing.
